# Expression of Thomsen–Friedenreich Antigen in Colorectal Cancer and Association with Microsatellite Instability

**DOI:** 10.3390/ijms22031340

**Published:** 2021-01-29

**Authors:** Beatriz Leão, Xiaogang Wen, Henrique O. Duarte, Irene Gullo, Gilza Gonçalves, Patrícia Pontes, Claudia Castelli, Francisca Diniz, Stefan Mereiter, Joana Gomes, Fátima Carneiro, Celso A. Reis

**Affiliations:** 1 Faculty of Medicine, University of Porto, 4200-319 Porto, Portugal; beatriz.farias.leao@gmail.com (B.L.); irene.gullo12@gmail.com (I.G.); fcarneiro@ipatimup.pt (F.C.); 2 i3S—Instituto de Investigação e Inovação em Saúde, Universidade do Porto, 4200-135 Porto, Portugal; hduarte@ipatimup.pt (H.O.D.); fdiniz@ipatimup.pt (F.D.); stefan.mereiter@imba.oeaw.ac.at (S.M.); joanag@ipatimup.pt (J.G.); 3 Department of Pathology, Centro Hospitalar Vila Nova de Gaia/Espinho, 4434-502 Vila Nova de Gaia, Portugal; wxiaogang@ipatimup.pt; 4 IPATIMUP—Institute of Molecular Pathology and Immunology, University of Porto, 4200-135 Porto, Portugal; gilzasofia@gmail.com (G.G.); pcpontes@gmail.com (P.P.); 5 Department of Pathology, Centro Hospitalar Universitário São João, 4200-319 Porto, Portugal; 6 Department of Pathology, Faculty of Medicine, University of Porto, 4200-319 Porto, Portugal; 7 Department of Diagnostics and Public Health, Section of Pathology, University and Hospital Trust of Verona, 37134 Verona, Italy; castelli911@gmail.com; 8 Instituto de Ciências Biomédicas Abel Salazar, Universidade do Porto, 4050-313 Porto, Portugal

**Keywords:** glycosylation, colorectal cancer, microsatellite instability, Thomsen–Friedenreich antigen, *O*-glycan

## Abstract

Microsatellite instability (MSI) is a molecular phenotype due to a deficient DNA mismatch repair (dMMR). In colorectal cancer (CRC), dMMR/MSI is associated with several clinical and histopathological features, influences prognosis, and is a predictive factor of response to therapy. In daily practice, dMMR/MSI profiles are identified by immunohistochemistry and/or multiplex PCR. The Thomsen–Friedenreich (TF) antigen was previously found to be a potential single marker to identify MSI-high gastric cancers. Therefore, in this study, we aimed to disclose a possible association between TF expression and MSI status in CRC. Furthermore, we evaluated the relationship between TF expression and other clinicopathological features, including patient survival. We evaluated the expression of the TF antigen in a cohort of 25 MSI-high and 71 microsatellite stable (MSS) CRCs. No association was observed between the expression of the TF antigen and MSI-high status in CRC. The survival analysis revealed that patients with MSI-high CRC showed improved survival when the TF antigen was expressed. This finding holds promise as it indicates the potential use of the TF antigen as a biomarker of better prognosis in MSI-high CRCs that should be validated in an independent and larger CRC cohort.

## 1. Introduction

Colorectal cancer (CRC) is a worldwide health burden disease, being the third most incident cancer and the second cause of cancer-related death [1]. Environmental factors such as diet, obesity, and sedentary behavior are risk factors for the development of CRC [2] that occurs via stepwise accumulation of genetic and epigenetic alterations [3]. The consensus molecular classification of CRC encompasses four distinct subtypes: Consensus Molecular Subtype (CMS) 1 (microsatellite instability, 14%), CMS2 (canonical, 37%), CMS3 (metabolic, 13%), and CMS4 (mesenchymal, 23%) [4]. Almost all hypermutated CRCs with microsatellite-instability (MSI-high) fall into the first category (CMS1). The microsatellite stable (MSS) cancers are subcategorized into the three other groups, CMS2 to CMS4, with a residual unclassified group (mixed features, 13%) that may represent either a transition phenotype or intratumoral heterogeneity. The hypermutated pathway is caused by a defect in the DNA mismatch repair (dMMR) mechanism and can be either sporadic (≈12%) or hereditary (≈3%) [5,6].

MSI is a molecular phenotype of tumors resulting from indel mutations in tandemly repeated nucleotide sequences present throughout the genome called microsatellites, caused by the impairment of the DNA mismatch repair (MMR) machinery. Major MMR genes encompass *MLH1*, *MSH2*, *MSH6*, and *PMS2* [7,8]. MSI status is a determining factor in CRC, influencing the clinical outcome [9], namely response to therapy and prognosis [9,10]. These factors are taken into consideration for planning the treatment of CRC patients, which requires a multidisciplinary approach [11]. Several studies have reported MSI-high as a predictive marker for the lack of response to fluorouracil-based adjuvant therapy in CRC, indicating that the efficacy of this type of chemotherapy differs according to MSI status [12,13]. A number of retrospective studies, including a systematic review and a meta-analysis, support the favorable stage-adjusted prognosis of MSI-high compared to MSS CRC patients [11,14,15,16,17].

The gastrointestinal mucosa is covered by a mucous layer rich in high extensive *O*-glycosylated proteins, called mucins, that constitute a protective layer over the epithelium [18]. In the process of carcinogenesis, several changes in the protein glycosylation machinery occur, resulting in aberrant cell surface glycosylation profiles [19]. These alterations are characterized by increased sialylation, fucosylation or truncation of O-glycans and are often observed in gastrointestinal tumors [19,20]. Moreover, previous studies have shown a correlation between altered glycans and tumor progression in gastrointestinal cancer [18,21,22,23,24,25,26,27]

One illustrative example of aberrant *O*-glycans signatures is the simple disaccharide antigen Thomsen–Friedenreich antigen (TF antigen)*,* also named antigen T or Core 1 [28]. This antigen is an intermediate product that appears in the Golgi apparatus during the maturation of mucin-type-*O*-glycans [19]. The TF antigen is rarely detected in normal cells but is frequently expressed in tumor cells, with pathological and clinical consequences [29]. Moreover, TF antigen expression in liver metastasis, the most common hematogenic dissemination in CRC, was described in a pilot study that demonstrated that CRC liver metastasis expressed the TF antigen at a significantly higher rate (91%) than in primary CRC (60%) [30]. For this reason, this truncated *O*-glycan arises as biomarker of malignancy with possible implications in the diagnosis, prognosis, and follow-up.

A previous study by Mereiter et al. [31] showed, in gastric cancer, a strong association between the expression of the TF antigen and the MSI-high status (specificity of 94% and sensitivity of 69%), suggesting that TF antigen is a single specific and sensitive marker for the MSI-high status in gastric cancer [31]. In the literature, there is no data on the association between the expression of TF antigen and the MSI status in CRC. Therefore, we aimed to perform an exploratory study to evaluate whether such an association is also present in CRC. Furthermore, we evaluated the association between TF expression and additional clinicopathological variables, including survival analysis. We performed histochemistry analysis using the Peanut agglutinin (PNA) lectin that preferentially binds to the galactosyl (β-1,3) *N*-acetylgalactosamine structure, the TF antigen [32], to evaluate if the detection of this biomarker could be a tool in the identification of MSI-high CRC.

## 2. Results

### 2.1. TF Expression in Colorectal Cancer

The expression of TF epitope, detected by histochemistry with PNA lectin, was evaluated in 96 colorectal carcinomas and detected in 55 cases (57%). Several parameters were assessed regarding the TF expression in CRC tumors: the percentage of labeled cancer cells, intensity of the staining, and the subcellular localization. The TF expression in the extracellular mucus secretion was also evaluated and recorded as “intraglandular” (in the lumen of glands), localized in “mucin pools” or both. (Supplementary Materials, Table S1). TF expression in the neoplastic cells was considered positive when more than 5% of tumor cells were stained.

**Figure 1:**
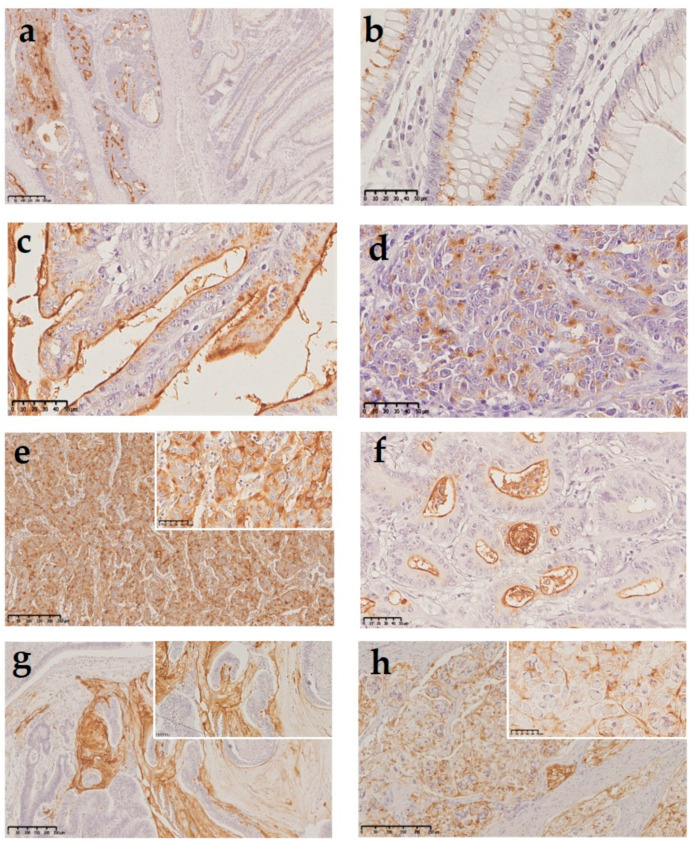
Thomsen–Friedenreich antigen (TF) expression in human colorectal tissue samples: (**a**) colorectal adenocarcinoma (left) and nonneoplastic mucosa adjacent to the tumor (right), both expressing the TF antigen (100× magnification); (**b**) high power of the nonneoplastic mucosa displaying perinuclear staining of the TF antigen in the Golgi apparatus (400× magnification); (**c**) strong expression in the apical membrane in a low-grade carcinoma (400× magnification); (**d**) cytoplasmatic staining in a high-grade carcinoma with a solid tumoral component (400× magnification); (**e**) cytoplasmatic and membranous staining in a high-grade adenocarcinoma with trabecular structure (200× magnification—insert 400×); (**f**) TF expression in intraglandular mucus in a low-grade adenocarcinoma (400× magnification); (**g**) TF expression in mucin pools in the mucinous component of a low-grade adenocarcinoma (50× magnification—insert 400×); and (**h**) TF expression in signet ring cells (100× magnification—insert 400×).

The TF antigen was observed in the nonneoplastic mucosa adjacent to the tumors (Figure 1a). In this localization, the antigen expressed a perinuclear staining in what appears to be the Golgi apparatus (Figure 1b). Low-grade carcinomas typically showed a strong ectopic expression in the apical membrane (Figure 1c). In contrast, high-grade carcinomas evidenced cytoplasmatic staining (Figure 1d) or both membranous and cytoplasmatic staining (Figure 1e). Regarding extracellular mucus staining, the TF expression was mainly positive in intraglandular mucus in low-grade carcinomas (Figure 1f) and mucin pools were marked prominently for the TF antigen, as shown in Figure 1g, in the mucinous component of a low-grade carcinoma. The TF expression was detected also in carcinomas with signet ring cells (Figure 1h).

The whole series (96 patients) consisted of 85 (89%) low-grade carcinomas and 11 (11%) high-grade carcinomas. The TF expression was analyzed according to the tumor grading of the tumor (Table S2), and we did not find any significant association with % and intensity of positive cells or with the type and location of intracellular and extracellular staining.

The whole series (96 patients) was also assessed to find possible associations between TF expression and clinicopathological variables (Table 1).

Concerning macroscopic type, we found a significant statistical association between TF expression and macroscopic type (*p* = 0.02), as ulcerated tumors showed more frequently TF expression. Regarding tumor desmoplasia, we found also a statistical association between the expression of TF and this feature, as TF positive tumors showed a moderate/strong desmoplasia than TF negative tumors (*p* = 0.04).

Regarding TNM staging, a significant statistical association between TF expression and T staging was found (*p* = 0.02), with higher TF expression in tumor progression (7% in pT1, 26% in pT2, and 51% in pT3).

Regarding other clinicopathological features evaluated in this cohort (Table 1), we did not find any statistically significant association with the TF status.

The MSI status was evaluated and compared with the expression of the TF antigen (Table 2). This cohort encompasses 25 cases (26%) classified as MSI-high and 71 cases (74%) as MSS. The expression of TF antigen was found in 15 (60%) MSI-high cases and 40 (56%) MSS cases (Table 2). No statistically significant association was found between the expression of TF antigen and the MSI status (*p* = 0.47). The sensitivity for MSI-high detection using TF antigen histochemistry was 60% (among the 25 MSI-high cases, only 15 had TF expression) and the specificity was 43.7% (among the 71 MSS cases, 31 did not have TF expression). The positive and negative predictive values were, respectively, 27.3% (15/55) and 75.6% (31/41).

### 2.2. Survival Analysis

**Figure 2:**
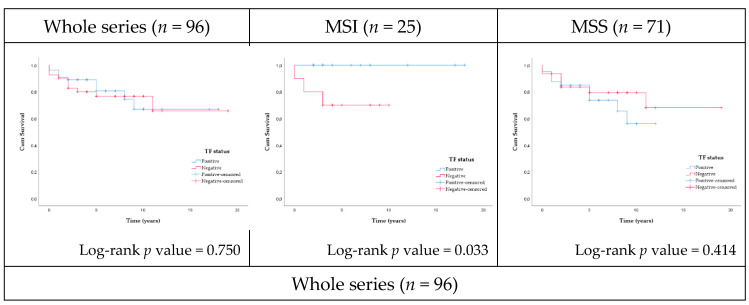

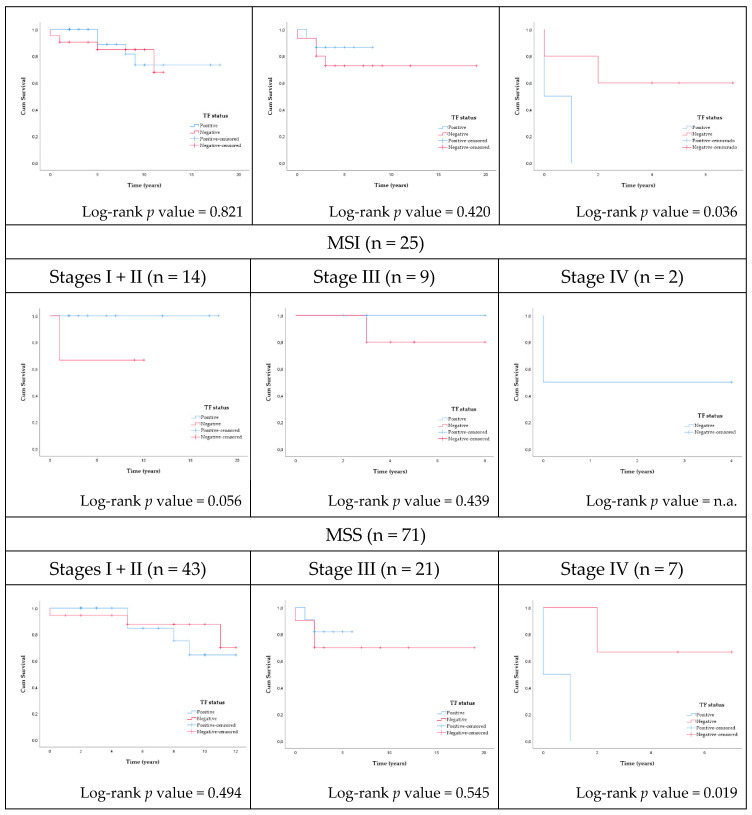
Kaplan–Meier curves of the overall survival of the patients according to TF expression in the tumors.

Survival was evaluated in the whole series and according to MSI status (MSI-high and MSS) and TNM stages (stages I + II, stage III, and stage IV) (Figure 2). In this exploratory cohort, the 5-year survival rate decreased with TNM stages: early stages (I/II)—88%, stage III—79%, and stage IV—33%, in keeping with data reported in the literature [33].

In the whole series, no association was found between TF expression and the overall survival of the patients. In the MSI-high subset, the survival of patients harboring TF-positive tumors was significantly better than in the negative cases (log rank *p*-value = 0.033). When MSI-high cases were stratified by TNM stage, within the patients harboring early stage (I + II) tumors, a better survival was observed in those with TF-positive tumors (log-rank *p* value = 0.056).

When the whole series was stratified by stage, a significant lower survival was observed in stage IV patients with TF-positive tumors (mean survival time 0.50 years) compared with stage IV patients harboring TF-negative tumors (mean survival time 3.60 years) (*p* = 0.036). Additionally, in stage IV MSS cases, the survival of patients harboring TF-positive tumors (mean survival time 0.50 years) was significantly lower than in TF-negative cases (mean survival time 4.67 years) (*p* = 0.019).

## 3. Discussion

Glycoconjugates are major components of the cell, playing important roles in various biological processes [34]. Altered glycosylation has been shown to influence cellular behavior, affecting and controlling numerous pathophysiological aspects of cancer, including progression [19], immune escape [35,36], tumoral invasion, and metastases [37]. The understanding of these mechanisms is fundamental and may contribute to the implementation of glycosylation modifications in clinical practice [38]. Aberrant cancer-associated glycans and glycoproteins have been used in the clinical context, mostly as serological markers. An example is the use as a biomarker of the carcinoembryonic antigen (CEA), a glycoprotein involved in cell adhesion [39]. It is overexpressed in carcinomas of the colon, rectum, breast, and lung [40]. In CRC, it is present in most patients, being used in the evaluation of prognosis and follow up, particularly after surgical resection [41]. In this study, we evaluated in situ the expression of TF antigen, a truncated *O*-glycan that has been reported to be expressed in different tumors, such as ovarian cancer, breast cancer, CRC, and acute lymphoblastic leukemia (T-cell) [42]. Regarding CRC, the TF antigen was found to be expressed in 60% of cases [43]. Concerning its role, the TF antigen has been implicated in cell adhesion, as it favors the attachment of tumor cells to the endothelium through the expression of galectin-3 by endothelial cells, supporting its role in tumor invasion [44,45] and therefore contributing to metastasis [30].

The predictive biomarkers used in clinical practice for CRC patients include mutations of the *NRAS*, *KRAS*, and *BRAF* genes as well as MSI status. Particularly, MSI status is relevant as a prognostic factor and predictive biomarker for therapy response.

MSI can be identified by two methods [9]: immunohistochemistry (IHC)-based detection of MMR proteins (MLH1, MSH2, MSH6, and PMS2) in tumor cells or molecular assays using polymerase chain reaction (PCR) or next-generation sequencing (NGS) for the evaluation of alterations in the microsatellites. Although both approaches provide reliable results for diagnostic purposes, these methods have some limitations [46]. MSI testing by multiplex PCR or NGS does not give information on which MMR gene may be involved, is not readily available in all laboratories, and has lower sensitivity than IHC in low tumor purity cases [46,47]. By contrast, IHC indicates which MMR gene may be abnormal, is generally available in all laboratories, and requires lower turnaround times. However, IHC may lead to less consistent results due to pre-analytic and analytic variables as well as interobserver variability [48]. Therefore, these limitations raise the interest in additional biomarkers to detect MSI in the clinical setting [49].

In this exploratory study, we identified 25 MSI-high cases (26%) and 71 MSS cases (74%). All cases were evaluated by both methods and only two discrepant cases (2%) were found. Both cases showed loss of MLH1 and PMS2 nuclear expression but were considered MSS by PCR testing. Possible explanations for the discordant results include tumoral heterogeneity [50] and/or underrepresentation of tumor cells in the sample [51,52]. We have a higher frequency of MSI-high cases in this series than reported in the literature (15–20%) [5]. The limited number of cases within this exploratory analysis highlights the importance of an independent cohort for further validation.

Currently, the treatment of CRC rests on two pillars: surgery and chemotherapy [53]. For therapeutic decision, MSI status has been pointed out as a factor that impacts clinical response to conventional treatments [11]. The decision of adjuvant therapy differs according to MSI status in stage II CRC in intermediate risk patients, as it is not a recommended adjuvant therapy in this group of patients [11]. It has become evident that the “one-size-fits-all” approach is no longer acceptable in the treatment of CRC that is evolving to a more personalized approach, taking into consideration the neoplastic genomic landscape that gained *momentum* in the treatment strategy. Moreover, immunotherapy is evolving at an enthusiastic speed in the field of oncology. In CRC with MSI, especially in metastatic chemorefractory MSI-high CRC [54], immunotherapy using PD-1 and PD-L1 checkpoint inhibitors is providing promising results regarding sustained clinical response [55] due to the fact that, in MSI-high tumors, there is upregulation of immune checkpoints [56].

Here, we present for the first time a study that correlates the expression of the TF antigen in CRC with MSI. Our data indicate that the TF antigen is not a predictor of MSI in CRC, contrary to what has been described in gastric cancer with MSI [31]. However, our results showed that patients harboring MSI-high tumors that express TF antigen have a significantly better survival than TF-negative cases. Taking into consideration the size of the sample in this exploratory study, this finding should be evaluated in the future in larger cohorts. This will be important to define the potential use of the TF antigen as a biomarker of better prognosis in MSI cases of CRC.

Implications of patient survival in CRC tumors harboring TF expression may be related to cancer immunity. Previous reports have shown higher sensitivity to natural killer (NK) cells towards human carcinoma cell lines expressing the TF antigen [57]. Moreover, it was proposed that the TF antigen also participates in the recognition of endogenous lectins expressed by the immune cells [58] and therefore modulate the immune response. Additionally, MSI-high tumors are characteristically more immunogenic due to a high production of mutated peptides that act as tumor-specific neoantigens that stimulate a more vigorous immune response, both adaptive and innate, leading to a better prognosis [59]. This antigen-driven immune response is mediated by the lymphocytic infiltrate that is observed in MSI-high CRC [60]. Therefore, the simultaneous TF expression and MSI status may contribute to better prognosis due to modulation of the immune response to malignant cells.

Overall, our finding holds promise as it indicates the potential use of the TF antigen as a biomarker of better prognosis in MSI CRC cases. However, further studies validating the obtained predictive results in independent and larger CRC cohorts are warranted in order to be considered for potential clinical application.

## 4. Materials and Methods

### 4.1. Patients Samples

The series included 96 colorectal carcinomas retrieved retrospectively from the archives of the Department of Pathology of Centro Hospitalar Universitário de São João (CHUSJ). The patients included in the study were diagnosed with CRC (from January 2001 to December 2018) in which immunohistochemistry for MMR proteins had been previously performed in order to evaluate the MMR deficient status of the tumors. Patients submitted to neoadjuvant therapy and with Lynch syndrome were excluded.

The detailed clinicopathologic features, including gender; age of diagnosis; tumor site; macroscopic type; World Health Organization (WHO) histological classification [61]; tumor grading; growth pattern (ulcerated versus infiltrative); amount of desmoplastic reaction; amount of inflammatory infiltrate; pTNM classification based on the AJCC/UICC TNM classification, 8th edition [62]; residual (R) tumour status, lymphatic and/or venous invasion; perineural invasion; Dukes classification [63]; Jass/Morson classification [64]; adjuvant therapy; and CRC family history were collected using the Database of the Department of Pathology and the Clinical Files system of CHUSJ.

The study, which included access to clinicopathological data, was approved by the ethics committee of CHUSJ (no. 366/19).

### 4.2. Immunohistochemistry for the Detection of MMR Proteins

The immunohistochemistry for MMR proteins (MLH1, MSH2, MSH6, and PMS2) was assessed by evaluating the presence of a nuclear staining pattern in the tumor cells and classified as (1) intact expression, when ≥10% of the tumor cells showed preserved nuclear expression, or (2) abnormal expression, when the tumor showed complete loss of expression, expression in <10% of tumor cells [65], weaker staining compared with the internal control, or abnormal staining in the nucleoli or nuclear membrane. The presence of an appropriate positive internal control, namely nuclear staining in stromal cells, was consistently verified and compared to the staining of tumor cells.

### 4.3. MSI Testing

Molecular testing was performed using the Idylla^TM^ MSI Test [66] in which 7 biomarkers (ACVR2A, BTBD7, DIDO1, MRE11, RYR3, SEC31A, and SULF2) were amplified via PCR for a downstream melting curve analysis. Then, the analysis software detected the mutation status of each biomarker by calculating a probability score (MSI score) derived from the melting curve analysis, expressing the probability of a melting pattern being the wild type or mutant. Consistent with previously established criteria [67] within the software, the detection of at least two mutated markers classified the sample as MSI-high. Otherwise, if less than two markers were mutated, the sample was classified as MSS.

### 4.4. Histochemistry Profiling of the TF Antigen

In this study, the expression of the TF antigen was assessed through staining with the PNA lectin [32].

A 3 µm section was prepared from one representative formalin-fixed paraffin-embedded (FFPE) block for each sample. Sections were deparaffinated, rehydrated, and endogenous peroxidases were inactivated with 3% hydrogen peroxide in methanol. Tissue sections were blocked for 30 min in room temperature with normal rabbit serum in phosphate-buffered saline (PBS) with 10% bovine serum albumin (BSA). Tissue sections were incubated with 2 µg/mL biotinylated PNA (Vector Labs’, Burlingame, CA, USA) in PBS supplemented with 0.1 mM CaCl_2_ and 0.01 mM of MnCl_2_ for 1 h at room temperature. Then, the sections were incubated with ABC (avidine-biotin peroxidase) for an additional 30 min at room temperature. Finally, the sections were stained by 3,3′-iaminobenzidine tetrahydrochloride (DAB) (Sigma Aldrich, St. Louis, MO, USA) and counterstained with Gill’s hematoxylin solution for nuclear contrast. The slides were mounted using Entellan solution and examined using a Zeiss Optical Microscope.

The criteria used to assess the positivity for TF antigen in the tumor was the presence of more than 5% of positive cancer cells. A semiquantitative evaluation of the percentage of positive cancer cells was applied for the following groups: 0% to ≤5% positive cancer cells, >5% to <50% positive cancer cells, and ≥50% positive cancer cells. TF expression in the neoplastic cells was considered positive when more than 5% of the tumor cells were stained. Staining intensity was classified as absent, weak, or strong. The intracellular staining was assessed as membranous, cytoplasmic, or both. The TF expression in the extracellular mucus secretion was also evaluated and recorded as “intraglandular” (in the lumen of glands), localized in “mucin pools” or both.

### 4.5. Statistical Analysis

Statistical analysis was performed with IBM SPSS STATISTICS (version 26.0 for Windows; SPSS, Chicago, IL, USA).

To assess the presence of an association with statistical significance between clinicopathological variables and TF expression (positive versus negative), Fisher’s Exact Test and Pearson chi-squared test were applied, as appropriate. For numerical variables with normal distribution, Independent Samples *t* Test was used, while for numerical variables without a normal distribution, Mann–Whitney–Wilcoxon test was used. All tests were two-sided, and differences were considered significant when *p* < 0.05.

## Figures and Tables

**Table 1:** Thomsen–Friedenreich (TF) expression and clinicopathological features.

	Categories	Total	TF Positive	TF Negative	*p*-Value ^1^
		*n* (%)	*n* (%)	*n* (%)	
		96 (100%)	55 (57%)	41 (43%)	
Gender	F	44 (46%)	24 (44%)	20 (49%)	0.38
	M	52 (54%)	31 (56%)	21 (51%)	
Age of diagnosis	Mean value (Years)	54.8	53.5	56.7	0.23
Tumor Site	Right Hemicolon	45 (47%)	24 (44%)	21 (51%)	0.24
	Left Hemicolon	39 (41%)	21 (38%)	18 (44%)	
	Rectum	9 (9%)	7 (13%)	2 (5%)	
	Colon NOS	3 (3%)	3 (5%)	0 (0%)	
WHO Classification	Adenocarcinoma NOS	80 (83%)	45 (82%)	35 (85%)	0.66
	Mucinous	15 (16%)	9 (16%)	6 (15%)	
	Undifferentiated	1 (1%)	1 (2%)	0 (0%)	
Macroscopic Type	Ulcerated	60 (62%)	40 (73%)	20 (49%)	0.02 *
	Vegetant	20 (21%)	9 (16%)	11 (27%)	
	Infiltrative	2 (2%)	2 (4%)	0 (0%)	
	Polypoid	14 (15%)	4 (7%)	10 (24%)	
Tumor grading	Low-grade	85 (88%)	49 (89%)	36 (88%)	0.55
	High-grade	11 (12%)	6 (11%)	5 (12%)	
R status	R0	91 (95%)	52 (95%)	39 (95%)	0.66
	R1	4 (4%)	2 (4%)	2 (5%)	
	R2	1 (1%)	1 (1%)	0 (0%)	
Growth pattern	Infiltrative	69 (72%)	37 (67%)	32 (78%)	0.18
	Expansive	27 (28%)	18 (33%)	9 (22%)	
Desmoplasia	Absent/Mild	38 (40%)	17 (31%)	21 (51%)	0.04 *
	Moderate/Strong	58 (60%)	38 (69%)	20 (49%)	
Inflammatory infiltrate	Absent/Mild	49 (51%)	27 (49%)	22 (54%)	0.41
	Moderate/Strong	47 (49%)	28 (51%)	19 (46%)	
pT (TNM Classification)	pT1	17 (18%)	4 (7%)	13 (32%)	0.02 *
	pT2	20 (21%)	14 (26%)	6 (15%)	
	pT3	43 (45%)	28 (51%)	15 (36%)	
	pT4	16 (16%)	9 (16%)	7 (17%)	
pN (TNM Classification)	pN0	57 (59%)	36 (65%)	21 (51%)	0.36
	pN1	28 (29%)	14 (26%)	14 (34%)	
	pN2	11 (12%)	5 (9%)	6 (15%)	
pM (TNM Classification)	M0	86 (90%)	51 (93%)	35 (85%)	0.20
	M1	10 (10%)	4 (7%)	6 (15%)	
Staging	Early (I & II)	57 (59%)	36 (66%)	21 (51%)	0.36
	III	30 (31%)	15 (27%)	15 (37%)	
	IV	9 (10%)	4 (7%)	5 (12%)	
Peritoneal Implants	Present	3 (3%)	2 (4%)	1 (2%)	0.61
	Absent	93 (97%)	53 (96%)	40 (98%)	
Lymphatic and/or venous invasion	Present	60 (62%)	36 (66%)	24 (58%)	0.32
	Absent	36 (38%)	19 (34%)	17 (42%)	
Perineural invasion	Present	29 (30%)	14 (26%)	15 (37%)	0.17
	Absent	67 (70%)	41 (74%)	26 (63%)	
Adjuvant Therapy	Performed	54 (56%)	30 (54%)	24 (58%)	0.43
	Not performed	42 (44%)	25 (46%)	17 (42%)	
Dukes classification	A	28 (29%)	13 (24%)	15 (37%)	0.07
	B	29 (30%)	22 (40%)	7 (17%)	
	C	38 (40%)	19 (34%)	19 (46%)	
	D	1 (1%)	1 (2%)	0 (0%)	
Jass/Morson classification	I	27 (28%)	16 (29%)	11 (27%)	0.99
	II	25 (26%)	14 (25%)	11 (27%)	
	III	25 (26%)	14 (26%)	11 (27%)	
	IV	19 (20%)	11 (20%)	8 (19%)	
CRC Family History	Present	26 (27%)	16 (29%)	10 (24%)	0.39
	Absent	70 (73%)	39 (71%)	31 (76%)	
Survival time	Mean value (Years)	5.4	4.8	6.1	0.09

Statistical significant results (*p* < 0.05) are marked with an asterisk (*). ^1^ Pearson chi-squared test, Fisher’s Exact Test, Independent Samples T Test (age of diagnosis), and Mann–Whitney–Wilcoxon test (survival time).

**Table 2:** Expression of TF antigen according to microsatellite instability (MSI) status.

	Categories	Total	TF Positive	TF Negative	*p*-Value ^1^
		*n* (%)	*n* (%)	*n* (%)	
		96 (100%)	55 (57%)	41 (43%)	
MSI status	High	25 (26%)	15 (27%)	10 (24%)	0.47
	Stable	71 (74%)	40 (73%)	31 (76%)	

^1^ Fisher’s Exact Test.

## Data Availability

Data sharing is not applicable to this article.

## Supplementary Materials

Supplementary materials can be found at https://www.mdpi.com/1422-0067/22/3/1340/s1.

## Abbreviations

ABC	Avidine-Biotin Peroxidase
BSA	Bovine serum albumin
CEA	Carcinoembryonic antigen
CHUSJ	Centro Hospitalar Universitário de São João
CMS	Consensus Molecular Subtype
CRC	Colorectal Cancer
DAB	3,3′-diaminobenzidine tetrahydrochloride
FFPE	Phosphate-buffered saline
IHC	Immunohistochemistry
MMR	Mismatch Repair
MSI	Microsatellite instability
MSS	Microsatellite stable
NGS	New generation sequencing
NK	Natural killer cells
PBS	Phosphate-buffered saline
PNA	Peanut agglutinin
PCR	Polymerase chain reaction
TF	Thomsen–Friedenreich antigen
WHO	World Health Organization
