# Supplementary material for: Expression of Thomsen–Friedenreich Antigen in Colorectal Cancer and Association with Microsatellite Instability
Source: Int J Mol Sci. 2021 Jan 29;22(3):1340. doi: 10.3390/ijms22031340 (PMC7866256; doi:10.3390/ijms22031340)
Supplement: Supplementary file 1 [file ijms-22-01340-s001.pdf]

Supplementary Materials

Table S1. Features of the TF expression in CCR tumours

	Total	TF Positive	TF Negative
	n (%)	n (%)	n (%)
	96 (100%)	55 (57%)	41 (43%)
**% of cell positivity**			
0% - ≤ 5%	41 (43%)	0 (0%)	41 (100%)
>5% - <50%	32 (33%)	32 (58%)	0 (0%)
≥50%	23 (24%)	23 (42%)	0 (0%)
**Staining intensity**			
Weak	7 (7%)	7 (13%)	0 (0%)
Strong	48 (50%)	48 (87%)	0 (0%)
Not applicable	41 (43%)	0 (0%)	41 (100%)
**Intracellular staining**			
Positive	55 (57%)	55 (100%)	0 (0%)
Negative	41 (43%)	0 (0%)	41 (100%)
**Localisation of intracellular staining**[1]			
Membranous	14 (26%)	14 (26%)	0 (0%)
Cytoplasmatic	3 (5%)	3 (5%)	0 (0%)
Both	38 (69%)	38 (69%)	0 (0%)
**Extracellular mucus staining**			
Positive	49 (51%)	49 (89%)	0 (0%)
Negative	47 (49%)	6 (11%)	41 (100%)
**Location of extracellular mucus staining**[2]			
Intraglandular	32 (65%)	32 (65%)	0 (0%)
Mucin pools	8 (16%)	8 (16%)	0 (0%)
Both	9 (19%)	9 (19%)	0 (0%)

[1] Only positive cases for the intracellular staining (n=55) were analysed.
[2] Only positive cases for the extracellular mucus staining (n=49) were analysed.

Table S2. TF expression according to tumour grading of the CRC tumours

	Total n (%)	Low-grade n (%)	High-grade n (%)	p-value[1]
	96 (100%)	85 (89%)	11 (11%)	
**% of cell positivity**				
0% - ≤ 5%	41 (43%)	36 (42%)	5 (46%)	0.90
>5% - <50%	32 (33%)	29 (34%)	3 (27%)	
≥50%	23 (24%)	20 (24%)	3 (27%)	
**Staining intensity**				
Weak	7 (7%)	7 (8%)	0 (0%)	0.42
Strong	48 (50%)	42 (50%)	6 (54%)	
Not applicable	41 (43%)	36 (42%)	5 (46%)	
**Intracellular staining**				
Positive	55 (57%)	49 (58%)	6 (54%)	0.55
Negative	41 (43%)	36 (42%)	5 (46%)	
**Location of intracellular staining[2]**				
Membranous	14 (26%)	14 (29%)	0 (0%)	0.18
Cytoplasmatic	3 (5%)	2 (4%)	1 (17%)	
Both	38 (69%)	33 (67%)	5 (83%)	
**Extracellular mucus staining**				
Positive	49 (51%)	45 (53%)	4 (36%)	0.24
Negative	47 (49%)	40 (47%)	7 (64%)	
**Location of extracellular mucus staining[3]**				
Intraglandular	32 (65%)	31 (69%)	1 (25%)	0.12
Mucin pools	8 (16%)	6 (13%)	2 (50%)	
Both	9 (19%)	8 (18%)	1 (25%)	

[1] Pearson Chi-squared test and Fisher's Exact Test.

[2] Only positive cases for the intracellular staining (n=55) were analysed.

[3] Only positive cases for the extracellular mucus staining (n=49) were analysed.
